# Burden of multidrug-resistant bacteria among HIV-positive individuals in Ethiopia: A systematic review and meta-analysis

**DOI:** 10.1371/journal.pone.0309418

**Published:** 2024-08-26

**Authors:** Muluneh Assefa, Azanaw Amare, Mitkie Tigabie, Getu Girmay, Abebaw Setegn, Yenesew Mihret Wondmagegn, Mebratu Tamir, Debaka Belete, Melak Aynalem, Teshome Belachew, Sirak Biset

**Affiliations:** 1 Department of Medical Microbiology, School of Biomedical and Laboratory Sciences, College of Medicine and Health Sciences, University of Gondar, Gondar, Ethiopia; 2 Department of Immunology and Molecular Biology, School of Biomedical and Laboratory Sciences, College of Medicine and Health Sciences, University of Gondar, Gondar, Ethiopia; 3 Department of Medical Parasitology, School of Biomedical and Laboratory Sciences, College of Medicine and Health Sciences, University of Gondar, Gondar, Ethiopia; 4 Department of Hematology and Immunohematology, School of Biomedical and Laboratory Sciences, College of Medicine and Health Sciences, University of Gondar, Gondar, Ethiopia; Hawassa University College of Medicine and Health Sciences, ETHIOPIA

## Abstract

**Background:**

Multidrug-resistant (MDR) bacteria are a significant cause of severe infections, particularly in human immunodeficiency virus (HIV)-positive individuals because of their weakened immunity. Since there was no previous pooled representative data regarding the MDR bacteria among HIV-positive individuals in Ethiopia, this systematic review and meta-analysis is required.

**Methods:**

This study was conducted based on the Preferred Reporting Items for Systematic Reviews and Meta-Analyses (PRISMA) guidelines. A literature search was performed using PubMed, Medline, EMBASE, Google Scholar, Hinari, Web of Science, Science Direct, and African Journals Online databases. Data were extracted using Microsoft Excel 2019 and analyzed using STATA version 11.0 software. A random-effects model was used to estimate the pooled effect size of outcome variables across studies with a 95% confidence interval. The I^2^ statistic was used to check for heterogeneity. The presence of publication bias was determined using a funnel plot and Egger’s test with a p-value < 0.05 evidence of statistically significant bias.

**Results:**

The pooled prevalence of MDR was 58.02% (95% CI: 46.32–69.73%) with high heterogeneity (I2 = 97.1%, (p < 0.001). In subgroup analysis, the highest multi-drug resistance was observed in the Oromia region (80.95%), patients with multiple infections (82.35%), and studies identified both Gram-positive and Gram-negative bacteria (61.45%). Furthermore, the pooled prevalence of MDR bacteria colonizing HIV-positive individuals was 48.76%. Regarding MDR species, *Enterococci* (77.41%) and *Pseudomonas spp*. (84.60%) were commonly identified in individuals with HIV infection.

**Conclusion:**

Our study indicates a high burden of MDR among HIV-positive individuals in Ethiopia. The Oromia region, HIV patients with multiple infections, *Pseudomonas spp*., and *Enterococci* showed the highest MDR in the subgroup analysis. Therefore, regional hospitals should implement strategies to tackle MDR such as vaccination program, appropriate use of antibiotics, and further study on the associated factors of MDR bacteria in HIV are required.

## Introduction

Starting from Alexander Fleming’s discovery of penicillin in 1928 till the present day, antimicrobial resistance (AMR) has been recognized as an ancient biological phenomenon, even as novel antimicrobial agents discovered and manufactured [1, 2]. The increased dissemination of AMR is threatening the world as it leads to serious illnesses and prolonged hospital admissions, an increase in healthcare costs, higher drug costs, and treatment failure [3]. It has been estimated that the burden of deaths by AMR may increase to 10 million each year by 2050, and it may claim around 2.4 million lives in Europe, North America, and Australia between 2015 and 2050 if effective action is not taken now [4].

Globally, drug-resistant infections contributed to 4.95 million deaths in 2019 and it is estimated that bacterial resistance was directly responsible for 1.27 million deaths [5]. In the World Health Organization (WHO) European region, an estimated 541,000 deaths are associated with bacterial AMR, and 133,000 deaths are attributable to bacterial AMR [6]. Above 1.05 million deaths were associated with AMR and 250,000 deaths were directly caused by bacterial resistance in the WHO African region [7]. In Ethiopia, 21,200 deaths were attributable to bacterial AMR and 85,300 deaths were associated with AMR in 2019 [8]. Lack of AMR surveillance, overuse of antimicrobials, clinical misuse of drugs, poor infection control practice, transmission of resistant pathogens in healthcare settings, prior antimicrobial use, poor healthcare contact, inadequate adherence to prescribed antibiotics, and the presence of underlying comorbid conditions are among the main factors that increase AMR [9, 10].

Multidrug-resistant (MDR) bacteria are defined as bacteria that are resistant to at least one antimicrobial agent from three or more antimicrobial classes [11]. Multi-drug resistance in bacteria is a significant concern in the healthcare industry, particularly in individuals infected with human immunodeficiency virus (HIV). The presence of MDR bacteria in HIV-positive individuals can be attributed to several factors, including a weakened immune system, frequent hospital admissions, clinic visits, and prolonged treatment with antimicrobials [12]. Overall, bacteria are one of the main causes of hospitalization and death in HIV-positive individuals; bacterial infection accounts for almost a third of all hospital admissions and a quarter of all deaths in these populations [13]. The most dangerous MDR bacteria that complicate the treatment of infections in HIV patients are extended-spectrum beta-lactamase-producing *Pseudomonas aeruginosa*, *Acinetobacter baumannii*, *Escherichia coli*, Methicillin-resistant *Staphylococcus aureus* (MRSA), and Vancomycin-resistant *Enterococci* [14].

According to previous studies [15–36], the magnitude of infection or colonization of MDR bacteria related to HIV infection varies in different regions of Ethiopia. For instance, the prevalence of MDR ranged from 12% in Addis Ababa [33] to 88.4% in Gondar, Northwest Ethiopia [20]. To the best of our knowledge, there is no reported data on the national burden of MDR bacteria among HIV-positive individuals in Ethiopia, which is important for implementation of antimicrobial stewardship and infection prevention programs to minimize further complications of HIV. Therefore, this systematic review and meta-analysis provided a comprehensive understanding of the current status of MDR bacteria among HIV-positive individuals in Ethiopia.

## Methods

### Study design and protocol registration

This systematic review and meta-analysis were performed as per the Preferred Reporting Items for Systematic Reviews and Meta-Analyses (PRISMA) guidelines [37] **(S1 Table)**. The protocol has been registered in the International Prospective Register of Systematic Reviews (PROSPERO) under the assigned number CRD42024498426.

### Literature search strategy

This meta-analysis focused on MDR bacterial pathogens that cause infection or colonize individuals with HIV infection. A COCOPOP (Condition, Context, and Population) paradigm was used to determine the suitability of the included studies for this meta-analysis. The prevalence of MDR bacteria comprised the study’s condition (CO), HIV-positive individuals as the population (POP), and Ethiopia served as the context (CO). The search included studies published before January 2024 and the last search was performed between January 10 to 20/2024. Eight electronic databases (PubMed, Medline, EMBASE, Google Scholar, Hinari, Web of Science, Science Direct, and African Journals Online) were searched to identify articles reporting the prevalence of MDR bacteria in HIV-positive individuals in Ethiopia. We used search terms alone and in combination with Boolean operators such as "OR" or "AND". An example of a PubMed search strategy used was as follows: (((((bacteria) OR (infection)) OR (colonization)) AND ((((((antimicrobial resistance) OR (antibiotic resistance)) OR (multidrug-resistant)) OR (multi-drug resistance)) OR (MDR)) OR (antibiogram))) AND ((((human immunodeficiency virus) OR (immunocompromised)) OR (HIV/AIDS)) OR (HIV))) AND (Ethiopia). A manual search was conducted for relevant papers in the references of the included studies and other reviews. The articles retrieved were imported into EndNote X9 bibliographic software manager (Clarivate Analytics, Philadelphia, PA, USA).

### Outcome of interest

The key outcome of interest in this study was the prevalence of MDR bacteria among HIV-positive individuals in Ethiopia, as described in the original study. Infection is described as the invasion and multiplication of pathogenic bacteria within a host organism [38]. Colonization refers to the establishment and persistence of bacteria in a host or a specific environment without necessarily causing harm or disease [39].

### Studies eligibility

Three independent reviewers (MA, SB, and AA) screened the titles and abstracts of the identified studies to determine their eligibility. Full-text articles were then assessed for eligibility, and any disagreements between the reviewers were resolved through discussion. Articles published regarding MDR in the English language with a cross-sectional study design that included the prevalence of MDR bacteria for colonization or infection with HIV status in the results fulfilled the current definition of MDR, were conducted in Ethiopia and without limit on sample type, type of infection, and study period. The studies had to be original research articles and the data had to be presented in a format that allowed for meta-analysis. Studies not reporting MDR data, unclear results, not fulfilled current MDR definition, case reports, systematic reviews, meta-analysis studies, and conducted on tuberculosis were excluded from the study.

### Quality assessment

After removing duplicated papers, all potentially eligible papers were reviewed. Full-text papers were retrieved for review and relevant information was extracted. The Joana Briggs Institute (JBI) critical appraisal checklist for simple prevalence was used to assess the quality of included studies [40]. This tool comprised nine questions. For each question, a score of 0 was assigned for ‘not reported’ or ‘not appropriate’ and 1 for ‘yes’. Then, the scores were summarized to obtain a total score ranging from 0 to 9. Based on the assigned points, articles were categorized as having a high (7–9), medium (5–7), or low (0–4) quality. Accordingly, articles with high (7–9) and medium (5–7) quality were included in the final analysis **(S2 Table)**. Three independent authors (MA, SB, and AA) assessed the quality of the studies, and any disagreement was solved by discussing it with the fourth author (DB).

### Data extraction

Data were extracted from each study using the designed tool in Microsoft Excel 2019 (Microsoft Corp., Redmond, WA, USA) by four independent authors (MA, SB, AA, and DB). Any ambiguity and difference during extraction were resolved through discussion. The data extracted from eligible studies were author name, year of publication, area in which the study was conducted, study design, type of sampling method, number of HIV-positive individuals, number of HIV-positive individuals on antiretroviral therapy (ART), sex (male), age group, type of infection or colonization, type of sample, number of MDR isolates, and type and number of bacterial species reported as MDR.

### Statistical analysis

Eligible data were extracted into Microsoft Excel 2019 and then exported to the STATA version 11.0 software for analysis. The pooled prevalence of MDR and 95% confidence intervals were visually displayed using a forest plot. Subgroup analysis was performed based on regions in the country, sampling method, year of study, type of infection or colonization, and MDR bacterial group or species. The heterogeneity of the included studies was evaluated using an index of heterogeneity (I^2^ statistic) value of 0% = no heterogeneity, ≤ 25% = low, 25%– 50% = moderate, 50–75 = substantial, and ≥ 75% = high [41]. In all pooled analyses, heterogeneity resulting from differences in effects across different studies was accounted for using a random-effects model. A sensitivity analysis of each study’s impact on the overall prevalence was also conducted. Publication bias was statistically investigated using Egger’s test [42] and visual inspection of funnel plots. A p-value < 0.05 in Egger’s test was considered as evidence of statistically significant publication bias.

## Results

### Literature search results

**Fig 1:**
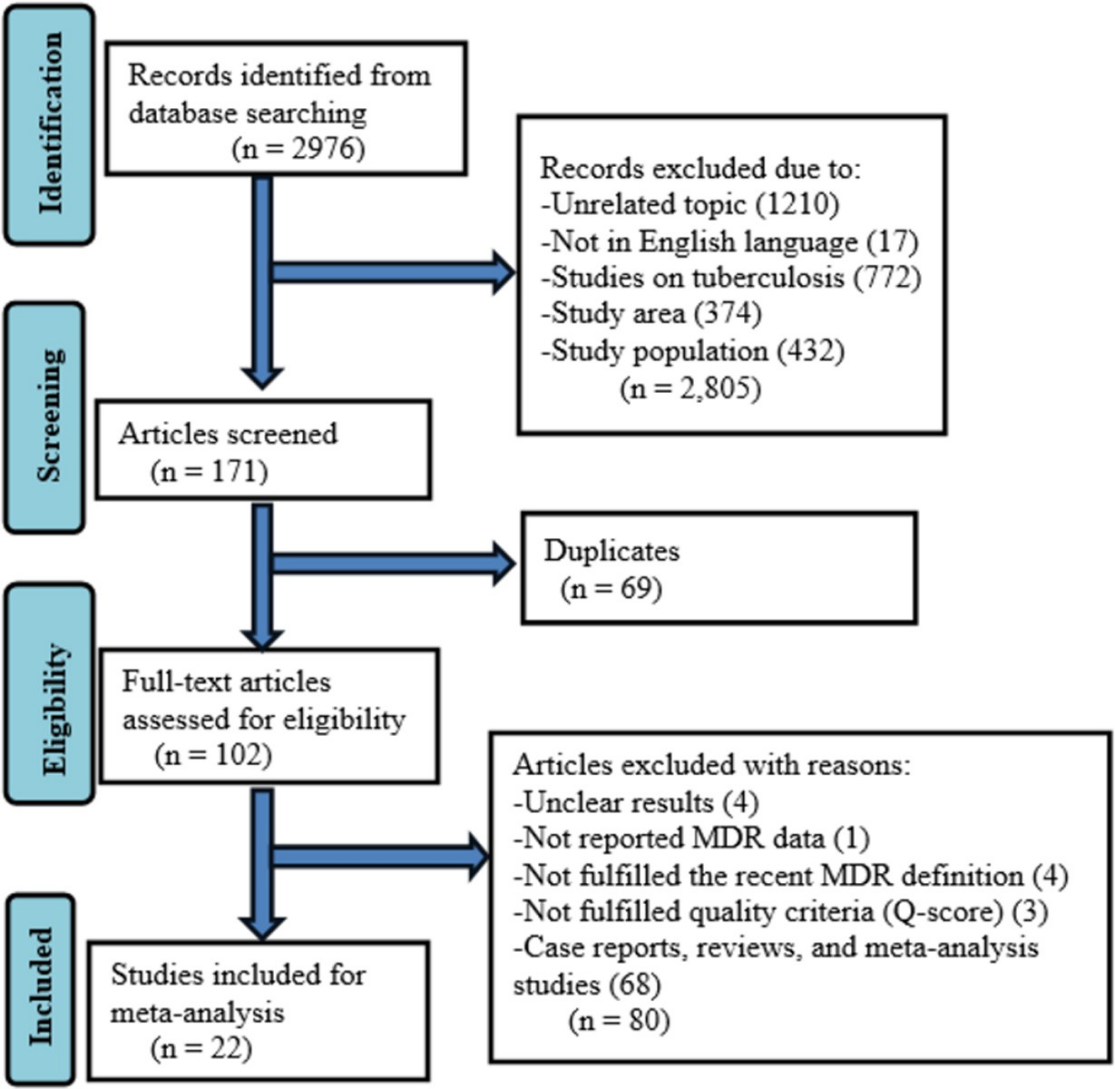
Flow diagram describing the selection of studies for the systematic review and meta-analysis on the burden of MDR bacteria among HIV-positive individuals in Ethiopia.

Initially, 2976 articles were identified in the literature search. The 171 were screened after removing articles for several reasons such as studies with unrelated topics, not written in the English language, studies on tuberculosis, study areas out of Ethiopia, and study populations other than HIV-infected individuals. Moreover, 69 duplicates were removed and 102 full-text articles were assessed for eligibility. Eighty full-text articles were excluded due to unavailable MDR data, not fulfilling the recent MDR definition, not fulfilling study quality criteria (Q-score), case reports, reviews, and meta-analysis studies. Finally, 22 studies were included in the meta-analysis **(Fig 1)**.

### Characteristics of the included studies

A total of 22 published articles [15–36] comprising 5636 HIV-positive individuals were included in this systematic review and meta-analysis. All of these studies used a cross-sectional study design. Regarding the sampling technique, 12 studies used systematic random sampling, 8 studies used convenient sampling, and the remaining 2 studies did not report their sampling techniques. Five regional states and one city administration in Ethiopia were represented by the studies: 10 from Amhara, 1 from Tigray, 7 from South Ethiopia, 1 from Sidama, 1 from Oromia, and 2 from Addis Ababa. The minimum and maximum numbers of study participants were 100 in Gondar [25] and 450 in Addis Ababa [21], respectively. Of the 5636 HIV-positive individuals, 3150 (55.9%) were females **(Table 1)**.

**Table 1:** Descriptive summary of studies on the prevalence of MDR bacteria among HIV-positive individuals in Ethiopia.

Author, year	Study region	Study year	Sampling method	HIV-positive individuals (N)	HIV-positive individuals on ART (%)	Sex (M)	Age group (Years)	Infection/colonization	Sample source	Total isolates (N)	MDR (N)	MDR group
Tilahun et al, 2023 [28]	Amhara	2021	Systematic random	378	100	187	≥ 10	Pneumonia	Sputum	175	148	GPB & GNB
Adhanom et al, 2019 [29]	Tigray	2016	NR	252	NR	127	≥ 18	Pneumonia	Sputum	84	15	GPB & GNB
Genetu& Zenebe, 2020 [32]	Amhara	2019	Convenient	163	100	62	≥ 18	Pneumonia	Sputum	68	53	GPB & GNB
Ayele et al, 2020 [23]	South Ethiopia	2019	Systematic random	180	NR	84	≥ 15	Diarrhea	Stool	15	11	GNB
Alebachew et al, 2016 [25]	Amhara	2013	Convenient	100	NR	12	all age	Bloodstream infection	Blood	31	25	GPB & GNB
Tilahun et al, 2023 [24]	Amhara	2021	Convenient	384	NR	199	all age	Multiple infections	Multiple samples	34	28	GPB
Tessema et al, 2020 [27]	Sidama	2018	Systematic random	224	NR	93	≥ 18	Urinary tract infection	Urine	23	18	GPB & GNB
Abebe et al, 2014 [20]	Amhara	2013	Systematic random	113	50.7	53	≥ 11	Colonization	Stool	103	91	GPB
Gebre et al, 2022 [33]	Addis Ababa	2016–18	NR	183	81.4	90	<15	Colonization	Nasopharyngeal swab	50	6	GPB
Bayleyegn et al, 2021 [30]	Amhara	2020	Convenient	161	100	77	< 15	Colonization	Stool	186	71	GNB
Jemal et al, 2020 [26]	Amhara	2018	Convenient	384	NR	157	all age	Bloodstream infection	Blood	123	96	GPB & GNB
Manilal et al, 2019 [16]	South Ethiopia	2017	Systematic random	307	NR	131	≥ 18	Colonization	Nasal swab	64	12	GPB
Dadi et al, 2021 [19]	South Ethiopia	2020	Systematic random	200	100	100	all age	Colonization	Stool	123	61	GPB
Fenta et al, 2016 [21]	Addis Ababa	2015	Convenient	450	71.0	141	≥ 18	Urinary tract infection	Urine	51	32	GPB & GNB
Simeneh et al, 2022 [22]	South Ethiopia	2021	Systematic random	251	NR	114	≥ 18	Urinary tract infection	Urine	39	21	GPB & GNB
Hantalo et al, 2020 [17]	South Ethiopia	2018	Systematic random	205	82.4	81	≥ 18	Urinary tract infection	Urine	29	23	GPB & GNB
Muhaba et al, 2022 [31]	Amhara	2020	Systematic random	206	100	77	all age	Colonization	Nasal swab	127	70	GPB
Seid et al, 2020 [18]	South Ethiopia	2018	Systematic random	252	95.6	144	≥ 15	Colonization	Nasal swab	34	10	GPB
Mulu et al, 2018 [15]	Amhara	2016–17	Convenient	300	NR	153	6–15	Colonization	Nasal and throat swab	167	47	GPB & GNB
Adisu et al, 2023 [34]	Oromia	2021	Convenient	351	100	135	≥ 18	Colonization	Nasal	21	17	GPB
Zike et al, 2024 [36]	Amhara	2023	Systematic random	170	NR	65	all age	Colonization	Stool	95	83	GPB
Mitiku et al, 2023 [35]	South Ethiopia	2022	Systematic random	422	NR	204	≥ 18	Diarrhea	Stool	63	31	GNB

**Note.** HIV: human immunodeficiency virus, ART: antiretroviral therapy, NR: not reported, MDR: multi-drug resistance, GPB: Gram-positive bacteria, GNB: Gram-negative bacteria, M: male, N: number.

### Prevalence of MDR among HIV-positive individuals

**Fig 2:**
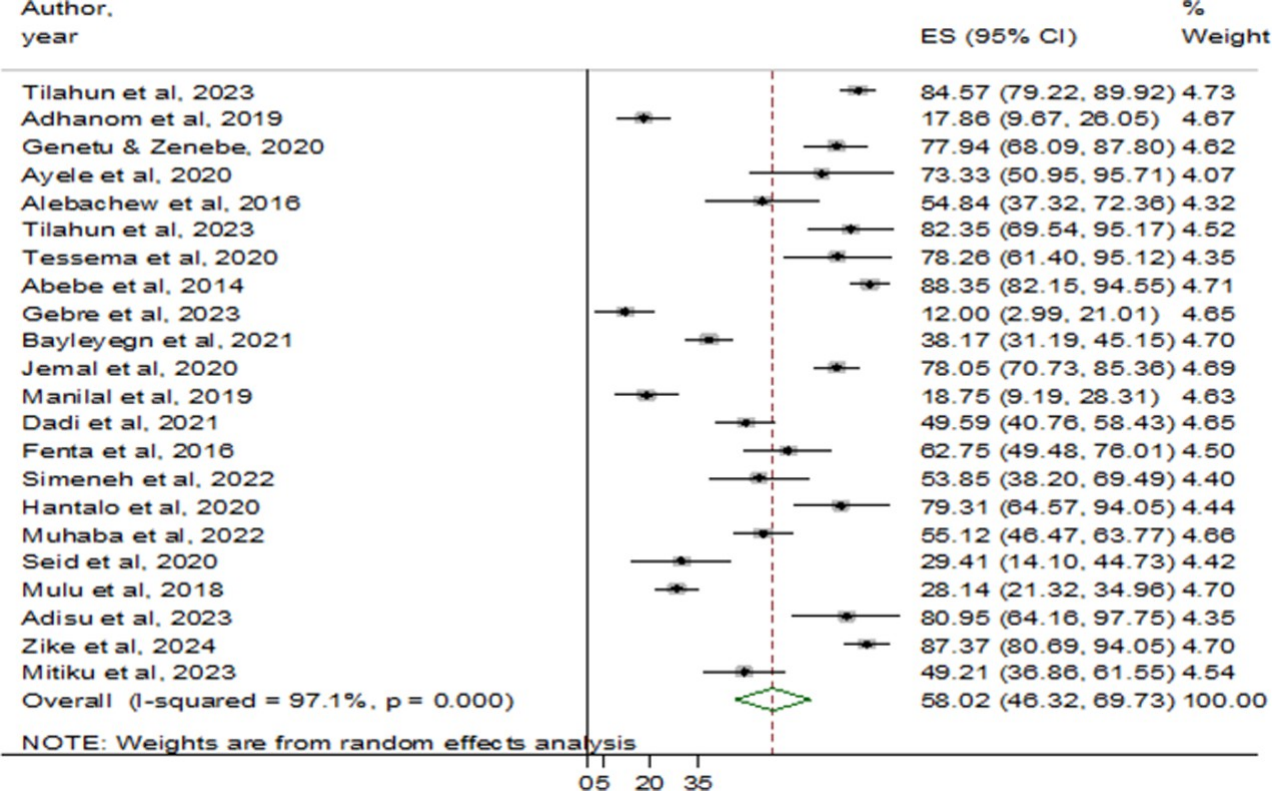
The forest plot showed the pooled prevalence of MDR bacteria among HIV-positive individuals in Ethiopia.

The minimum and maximum MDR prevalence reported by the studies was 12% (Addis Ababa) [33] and 88.4% (Gondar) [20], respectively. There were 1705 bacterial isolates identified from the eligible studies, of which 961 were MDR isolates **(Table 1)**. Accordingly, the pooled prevalence of MDR was 58.02% (95% CI: 46.32–69.73), with high heterogeneity (I^2^ = 97.1%) and statistical significance (p < 0.001) **(Fig 2)**.

### Subgroup analysis

The prevalence of MDR among different types of infection or colonization, regions of the studies performed, MDR species, years of the study, and sampling method were analyzed by subgroup analysis. The pooled prevalence of MDR per year of study was as follows: 2013–15; 69.66% (95% CI: 47.26–92.06%), 2016–19; 48.83% (95% CI: 29.95–67.72%), and 2020–23; 64.53% (95% CI: 50.32–78.74%). According to sampling methods, the MDR prevalence was described as systematic random; 62.32% (95% CI: 48.57–76.07%), convenient sampling; 62.61% (95% CI: 45.51–79.72%), and studies have not reported the sampling method; 15.21% (95% CI: 9.15–21.27%). The combined prevalence of MDR among different regions showed that the highest MDR was reported in Oromia; 80.95% (95% CI: 64.16–97.75%), followed by Sidama; 78.26% (61.40–95.12%), and Amhara; 67.57% (95% CI: 52.70–82.43%) **(Table 2)**.

**Table 2:** Subgroup analysis in the prevalence of MDR bacteria among HIV-positive individuals in Ethiopia.

Characteristics	No. of Studies	Total isolates	MDR	Estimated pooled prevalence of MDR (95% CI)	Heterogeneity		Egger’s test
					I^2^ (%)	P-value	
MDR in HIV	22	1705	961	58.02 (46.32–69.73)	97.1	< 0.001	0.532
Study year							
2013–15	3	185	140	69.66 (47.26–92.06)	90.6	< 0.001	
2016–19	10	657	287	48.83 (29.95–67.72)	97.1	< 0.001	
2020–23	9	824	509	64.53 (50.32–78.74)	95.6	< 0.001	
Sampling method							
Systematic random	12	890	579	62.32 (48.57–76.07)	96.0	< 0.001	
Convenient	8	681	361	62.61 (45.51–79.72)	95.9	< 0.001	
Not reported	2	134	21	15.21 (9.15–21.27)	0.0	0.346	
Region							
Amhara	10	1109	704	67.57 (52.70–82.43)	97.3	< 0.001	
Tigray	1	84	15	17.86 (9.67–26.05)	NA	NA	
Addis Ababa	2	101	38	37.13 (-12.60–86.86)	97.4	< 0.001	
South Ethiopia	7	367	169	49.70 (33.89–65.51)	90.4	< 0.001	
Sidama	1	23	18	78.26 (61.40–95.12)	NA	NA	
Oromia	1	21	17	80.95 (64.16–97.75)	NA	NA	
Infection/colonization							
Pneumonia	3	327	216	60.15 (17.84–102.46)	98.9	< 0.001	
Diarrhea	2	78	42	59.39 (36.03–82.75)	70.8	0.064	
Urinary tract infection	4	142	94	68.33 (56.48–80.18)	59.6	0.059	
Bloodstream infection	2	154	113	67.86 (45.29–90.44)	82.6	0.017	
Multiple infections	1	34	28	82.35 (69.54–95.17)	NA	NA	
Colonization	10	970	489	48.76 (30.14–67.38)	98.0	< 0.001	
MDR group							
GPB & GNB	10	790	470	61.45 (43.68–79.21)	97.1	< 0.001	
GPB	9	591	378	55.99 (35.28–76.70)	97.8	< 0.001	
GNB	3	264	113	50.53 (34.20–66.86)	79.7	0.007	

**Note.** HIV: human immunodeficiency virus, NR: not applicable, MDR: multi-drug resistance, GPB: Gram-positive bacteria, GNB: Gram-negative bacteria, CI: confidence interval.

Regarding infection or colonization, a higher combined prevalence of MDR bacteria was observed in HIV-positive individuals with multiple infections; 82.35% (95% CI: 69.54–95.17%), bloodstream infection (BSI); 67.86% (95% CI: 45.29–90.44%), pneumonia; 60.15% (95% CI: 17.84–102.46%), diarrhea; 59.39% (95% CI: 36.03–82.75%), and colonization; 48.76% (95% CI: 30.14–67.38%). Furthermore, the pooled prevalence of MDR in HIV-positive individuals per MDR group was 61.45% (95% CI: 43.68–79.21%) in studies identifying both Gram-positive and Gram-negative bacterial isolates, 55.99% (95% CI: 35.28–76.70%) in only Gram-positive bacteria, and 50.53% (95% CI: 34.20–66.86%) in Gram-negative bacteria **(Table 2)**.

### Pooled prevalence of MDR bacterial species

Of the included studies, the one conducted in Wolayta Sodo [17] did not report the type of bacterial species with MDR status was excluded. Bacterial species were included in the meta-analysis if reported by at least three studies. According to the type of bacterial species, four species of gram-positive bacteria and ten species of gram-negative bacteria were included in the meta-analysis. *S*. *aureus*, *Enterococci*, *S*. *pneumoniae*, and *E*. *coli* were the top four frequently identified isolates. The pooled proportion of MDR for each bacterial species was computed from the total isolates as summarized below **(Tables 3 and 4)**.

**Table 3:** Summary on the estimate of MDR for gram-positive bacteria in HIV-positive individuals.

Bacterial species	Estimate (95% CI) by infection and colonization		Overall pooled estimate (95% CI)	Heterogeneity	
	Infection	Colonization		I^2^ (%)	p-value
*S*. *aureus*	64.41 (37.72–91.10)	50.06 (13.10–87.03)	57.72 (40.77–74.68)	93.1	< 0.001
CoNS	74.58 (49.05–100.11)	-	74.58 (49.05–100.11)	30.0	0.232
*Enterococci*	81.79 (68.22–95.36)	75.95 (57.04–94.85)	77.41 (63.24–91.58)	87.2	< 0.001
*S*. *pneumoniae*	84.43 (74.37–94.50)	19.98 (-2.16–42.12)	50.51 (14.12–86.90)	86.5	< 0.001

**Note.** CI: confidence interval, CoNS: Coagulase-negative *Staphylococcus aureus*.

**Table 4:** Summary on the estimate of MDR for gram-negative bacteria in HIV-positive individuals.

Bacterial species	Estimate (95% CI) by infection and colonization		Overall pooled estimate (95% CI)	Heterogeneity	
	Infection	Colonization		I^2^ (%)	p-value
*E*. *coli*	68.27 (52.33–84.22)	41.44 (27.21–55.68)	62.39 (45.30–79.49)	69.7	0.003
*Klebsiella spp*.	65.11 (41.10–89.11)	26.47 (-2.35–55.29)	58.89 (34.40–83.39)	88.3	< 0.001
*Proteus spp*.	52.94 (5.41–100.48)	75.00 (26.00–124.00)	63.64 (29.52–97.75)	0.0	0.727
*Citrobacter spp*.	66.47 (39.24–93.70)	44.44 (-4.25–93.14)	61.70 (38.36–85.04)	0.0	0.447
*Acinetobacter spp*.	20.00 (-58.40–98.40)	-	20.00 (-58.40–98.40)	NA	NA
*Enterobacter spp*.	73.37 (47.26–99.49)	16.67 (-34.98–68.32)	57.11 (25.47–88.74)	36.1	0.196
*Pseudomonas spp*.	84.60 (71.95–97.24)	-	84.60 (71.95–97.24)	0.0	0.584
*Salmonella spp*.	63.01 (40.07–85.95)	57.14 (8.65–105.64)	61.94 (41.20–82.68)	0.0	0.971
*Shigella spp*.	38.71 (11.15–66.27)	33.33 (-32.00–98.67)	37.90 (12.51–63.29)	0.0	0.882
*H*. *influenzae*	77.78 (46.98–108.58)	33.33 (-32.00–98.67)	65.28 (26.11–104.44)	31.3	0.228

**Note.** CI: confidence interval, NA: not applicable.

### Multi-drug resistance in Gram-positive bacteria

In this meta-analysis, bacterial isolates from three genera (*Staphylococcus*, *Streptococcus*, and *Enterococci*) have been included. Among the included species, the pooled proportion of MDR for *S*. *aureus* was found to be 64.41% (95% CI: 37.72–91.10%) for infection and 50.06% (95% CI: 13.10–87.03%) for colonization **(Table 3)**. This study yielded an overall estimated MDR of 57.72% (95% CI: 40.77–74.68%) in *S*. *aureus* isolates. The estimate of MDR in Coagulase-negative *Staphylococci* (CoNS) was 74.58% (95% CI: 49.05–100.11%) for infection only. Concerning *Enterococci*, the pooled proportion of MDR was 81.79% (95% CI: 68.22–95.36%) for infection and 75.95% (95% CI: 57.04–94.85%) for colonization. The overall estimated MDR in *Enterococci* was 77.41% (95% CI: 63.24–91.58%). In the case of *S*. *aureus*, *Enterococci*, and *S*. *pneumoniae*, there was significant heterogeneity between the subgroups (infection and colonization) **(Table 3) (S1 Fig.)**.

### Multi-drug resistance in Gram-negative bacteria

Bacterial isolates belonging to the family *Enterobacteriaceae*, other gram-negative enteric bacteria, and genus *Haemophilus* have been included. The highest pooled MDR was observed in *Pseudomonas spp*. 84.60% (95% CI: 71.95–97.24%), followed by *H*. *influenzae* 65.28% (95% CI: 26.11–104.44%), *Proteus spp*. 63.64% (95% CI: 29.52–97.75%), and *E*. *coli* 62.39% (95% CI: 45.30–79.49%). In the case of *E*. *coli* and *Klebsiella spp*., there was higher heterogeneity between the subgroups (infection and colonization) **(Table 4)**. The pooled proportion of MDR for each bacterial species and group differences are described in the forest plots **(S1 Fig.)**.

### Publication bias

**Fig 3:**
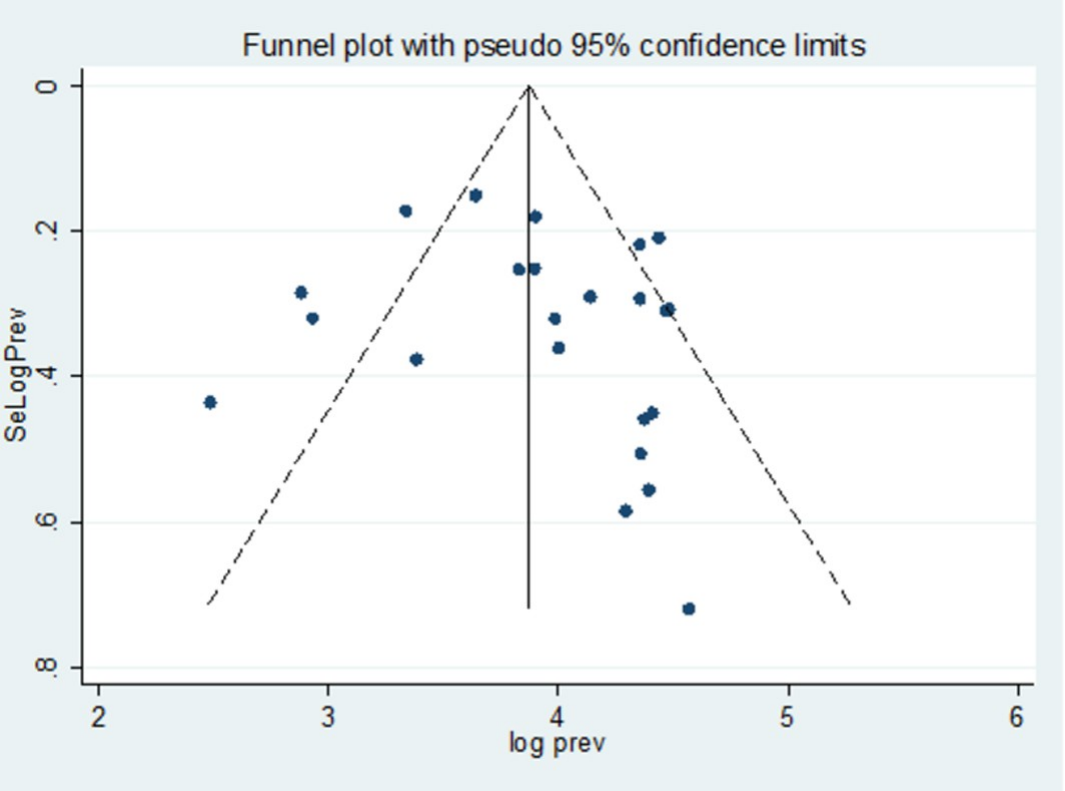
The funnel plot indicated publication bias in the studies on the burden of MDR bacteria among HIV-positive individuals in Ethiopia.

Studies were assessed for potential publication bias statistically using Egger’s test and funnel plot. The result of Egger’s test indicated no publication bias, -2.26 (95% CI: -9.68, 5.16, p-value = 0.532) **(Table 5)**. This was depicted graphically by a funnel plot, which showed a symmetrical display of prevalence reported by the studies **(Fig 3)**.

**Table 5:** Publication bias using Egger’s test.

Egger’s test					
Std_Eff	Coef.	Std. Err.	t	p>t	[95% Conf. Interval]
Slope	69.46217	16.6217	4.18	0.000	34.78991 104.1344
bias	-2.261594	3.557294	-0.64	0.532	-9.681979 5.158791

### Sensitivity analysis

A sensitivity analysis was performed using a random effects model because of the results’ extreme heterogeneity. Step-by-step removal of each study was used in a sensitivity analysis to determine how each study affected the pooled prevalence. The results showed that the omitted studies do not have a significant effect on the pooled prevalence of MDR among HIV-positive individuals **(Table 6).**

**Table 6:** Sensitivity analysis of the included studies to estimate the prevalence of MDR bacteria among HIV-positive individuals in Ethiopia.

Study omitted	Estimate (95% confidence interval)	Heterogeneity	
		I^2^ (%)	p-value
Tilahun et al, 2023	56.71 (44.74–68.68)	96.8	< 0.001
Adhanom et al, 2019	59.98 (48.54–71.43)	96.8	< 0.001
Genetu & Zenebe, 2020	57.06 (44.93–9.20)	97.2	< 0.001
Ayele et al, 2020	57.34 (45.38–69.38)	97.2	< 0.001
Alebachew et al, 2016	58.17 (46.11–70.23)	97.2	< 0.001
Tilahun et al, 2023	56.88 (44.84–68.92)	97.2	< 0.001
Tessema et al, 2020	57.12 (45.08–69.14)	97.2	< 0.001
Abebe et al, 2014	56.52 (44.69–68.34)	96.8	< 0.001
Gebre et al, 2022	60.25 (48.93–71.58)	96.7	< 0.001
Bayleyegn et al, 2021	59.01 (46.84–71.18)	97.1	< 0.001
Jemal et al, 2020	57.05 (44.81–69.23)	97.1	< 0.001
Manilal et al, 2019	59.92 (48.28–71.57)	96.9	< 0.001
Dadi et al, 2021	58.44 (46.17–70.72)	97.2	< 0.001
Fenta et al, 2016	57.81 (45.68–69.94)	97.2	< 0.001
Simeneh et al, 2022	58.22 (46.14–70.30)	97.2	< 0.001
Hantalo et al, 2020	57.04 (44.99–69.08)	97.2	< 0.001
Muhaba et al, 2022	58.18 (45.85–70.50)	97.2	< 0.001
Seid et al, 2020	59.35 (47.38–71.31)	97.2	< 0.001
Mulu et al, 2018	59.50 (47.74–71.24)	96.8	< 0.001
Adisu et al, 2023	56.98 (44.97–69.00)	97.2	< 0.001
Zike et al, 2024	56.57 (44.66–68.50)	96.9	< 0.001
Mitiku et al, 2023	58.45 (46.32–70.58)	97.2	< 0.001

## Discussion

In recent years, the prevalence of MDR bacteria among HIV-positive individuals has become a significant concern for healthcare providers and public health [13]. This systematic review and meta-analysis is the first to determine the pooled prevalence of MDR bacteria in HIV-positive individuals. The results included different types of bacterial infections and colonization in HIV-positive individuals in all age groups. Twenty-two studies based on 5636 HIV-positive individuals were included in this study. The results showed that the pooled prevalence of MDR bacteria among HIV-positive individuals was 58.02% (95% CI: 46.32–69.73%). Our finding is lower than systematic review and meta-analysis studies conducted in Ethiopia, which reported a 70.5% [43], 70.56% [44], 74.0% [45], and 74.2% [46] pooled prevalence of MDR bacteria. This might be due to the difference in types of infections, type of bacterial isolate, and study populations, which may increase the prevalence whereas our study was specific to HIV-positive individuals. Although there is no systematic review or meta-analysis conducted previously elsewhere focused on HIV-positive individuals, our finding indicated the higher burden of MDR. This might be due to the weakened immune system that leads to increased susceptibility to MDR bacterial infections, exposure to antibiotics, inadequate adherence to ART, and poor access to healthcare especially in resource-poor settings [47, 48]. Moreover, HIV infection alters the composition of the microbiome, which can create an environment conducive to the growth and persistence of MDR bacteria [49].

Our study indicates high heterogeneity (I^2^ = 97.1%) between the studies. Subgroup analysis was conducted to provide further details on the cause of this disparity. Accordingly, the prevalence of MDR in the different regions showed a statistically significant difference ranging from 80.95% to 17.86% with the highest and lowest pooled prevalence from Oromia and Tigray regions, respectively. The difference in number of studies between the regions could affect the MDR prevalence. This systematic review and meta-analysis included a single study from the Tigray [29], Sidama [27], and Oromia [34] regions based on our inclusion criteria. Most of the studies included were from Amhara, followed by South Ethiopia. This suggests that there are laboratory facilities for bacterial culture and susceptibility testing in these regions compared with others. In addition, the heterogeneity may be due to the few studies that were conducted on types of infection such as multiple infections [24], diarrhea [23, 35], and BSI [25, 26], a single study in the study period 2013–2015, and variations in the type and number of MDR bacteria identified between the studies.

The subgroup analysis showed that the prevalence of MDR bacteria among HIV-positive individuals across all regions of Ethiopia was higher in Oromia, followed by Sidama, Amhara, South Ethiopia, Addis Ababa, and Tigray. This variability may be due to differences in the healthcare system, the burden of infectious disease in a community, and the characteristics of the study population. The prevalence of MDR among HIV-positive individuals shows a sudden increment across different categories of study years as it is 69.66% and 64.53% in 2013–15 and 2020–23, respectively. The emergence of drug-resistant organisms, new resistance mechanisms, and a decrease in the efficiency of antibiotics treating antibiotic-resistant bacteria change the MDR prevalence over time [50].

According to the type of infection, a higher prevalence of MDR bacteria among HIV-positive individuals was observed in multiple infections, followed by urinary tract infection (UTI), BSI, pneumonia, and diarrhea. The prevalence of MDR bacteria in UTI is higher because most study participants in our study were females, who have a high risk of drug drug-resistant bacteria than males because of the structure of the urethra and vagina, and sexual activity facilitating pathogen entry [51]. Higher MDR in BSI is due to co-infections and chronic inflammation, a common feature of HIV infection, which can contribute to the development of bacterial bloodstream infections by damaging the lining of blood vessels [52]. Bacterial infections such as diarrhea and pneumonia are also common problems among HIV-positive individuals due to the immune system’s inability to effectively combat bacterial infections allowing opportunistic pathogens, such as certain MDR bacteria, to thrive and cause infections in the body [53, 54].

The meta-analysis showed that the prevalence of MDR bacterial colonization in HIV-positive individuals is somehow higher, with a pooled prevalence of 48.76%, and *Enterococci* were identified as highly colonizing bacteria in the studies. A previous systematic review and meta-analysis reported that HIV-positive individuals had 2.12 higher odds for colonization and 1.90 higher odds for infection with MRSA [13]. Another study conducted on the global prevalence of MRSA colonization in HIV-positive individuals reported a pooled prevalence of 7.0% [55]. This highlights the importance of monitoring and managing MDR bacterial colonization in HIV-positive individuals, particularly those with advanced immunosuppression. The colonizing bacterial flora in HIV-positive individuals are intrinsic sources of opportunistic infections and antibiotic-resistance genes can be transferred from such commensals to pathogenic organisms [56].

In this study, the highest pooled proportion of MDR among Gram-positive bacteria was detected in *Enterococci*, followed by CoNS and *S*. *aureus*. In addition, *Pseudomonas spp*., followed by *H*. *influenzae*, *Proteus spp*., and *E*. *coli* were the top MDR gram-negative bacteria. However, there are certain pieces of evidence of the pooled prevalence of MDR bacterial species in Ethiopia. A systematic review and meta-analysis data showed that the common multidrug-resistant species of bacteria from human, animal, food, and environmental sources were *S*.*aureus*, CoNS, *Pseudomonas spp*., *E*.*coli*, *Citrobacter spp*., *Klebsiella spp*., *Enterobacter spp*., and *Salmonella spp*. [57]. Another study on Gram-negative bacteria in wound infections reported that the pooled estimate of MDR in *Citrobacter spp*., followed by *K*. *pneumoniae*, *P*. *mirabilis*, *E*. *coli*, *Acinetobacter spp*., and *P*. *aeruginosa* [58]. Moreover, a study on pregnant women with bacteriuria revealed the top four MDR bacteria such as *E*. *coli*, *Klebsiella spp*, *S*. *aureus*, and CoNS [59]. This variability of some bacterial species and MDR prevalence is due to the difference in study population, type of infection, source of specimen, and type of antibiotics tested. Several factors contribute to the existence of MDR bacterial species in individuals with HIV infection, including frequent hospital visits, prolonged use of prophylaxis, and the risk of MDR bacteria prevalent in hospital settings [60]. In addition, this MDR occurred among these bacterial species because of the transfer of resistance genes, less permeability of the lipopolysaccharide layer, expression of efflux pumps, secretion of degrading enzymes, alteration of the target site, extreme use of broad-spectrum antibiotics, and scarcity of target-oriented antibiotics [61].

### Strengths

This systematic review and meta-analysis was the first report on the burden of MDR bacteria on HIV-positive individuals in Ethiopia. The strength of the study is its comprehensive literature search by three independent authors (MA, SB, and AA) to extract all available published articles. This study also focused on different types of bacterial infections and colonization in HIV-positive individuals.

### Limitations

In this study, we included only Amhara, Tigray, Addis Ababa, Oromia, Sidama, and South Ethiopia regions suggesting that they may not accurately generalize the public health problem in the country. Due to the lack of information in the published articles, viral load, and CD4 count were not fully reported in our meta-analysis.

## Conclusion and recommendations

This study highlights the alarming burden of MDR bacteria among HIV-positive individuals in Ethiopia with the common MDR species of *Enterococci*, CoNS, *S*. *aureus*, and *Pseudomonas spp*. A significant heterogeneity between the included studies and higher MDR prevalence in the Oromia, Sidama, and Amhara regions was observed. Therefore, regional hospitals should implement strategies to reduce antibiotic overuse and misuse, improve adherence to ART, optimize immune reconstitution, enhance access to healthcare services, further study on the complex relationship between HIV and the human microbiome, identifying risk factors of MDR in HIV, better access to diagnostic tools and antimicrobial stewardship, and early identification of opportunistic infections and colonization of bacteria in these regions with high MDR burden and all over the country is needed. Furthermore, the development of novel antimicrobial agents targeting MDR bacteria and vaccines for their prevention in HIV-positive individuals is crucial.

## Supporting information

S1 Table PRISMA checklist. (DOCX)

S2 Table Quality assessment of the studies included in systematic review and meta-analysis on the burden of MDR bacteria among HIV-positive individuals in Ethiopia. (DOCX)

S1 Fig Forest plots showed the pooled prevalence of MDR by each bacterial species among HIV-positive individuals in Ethiopia. (DOCX)

## Abbreviations

HIV: Human immunodeficiency virus
MDR: multidrug-resistant/ce
ART: Antiretroviral therapy
MRSA: Methicillin-resistant *Staphylococcus aureus*
BSI: Bloodstream infection
UIT: Urinary tract infection
PRISMA: Preferred Reporting Items for Systematic Reviews and Meta-Analyses
PROSPERO: International Prospective Register of Systematic Reviews
WHO: World Health Organization.
