# Supplementary material for: Burden of multidrug-resistant bacteria among HIV-positive individuals in Ethiopia: A systematic review and meta-analysis
Source: PLoS One. 2024 Aug 26;19(8):e0309418. doi: 10.1371/journal.pone.0309418 (PMC11346931; doi:10.1371/journal.pone.0309418)
Supplement: S2 Table — (DOCX) [file pone.0309418.s002.docx]

**Table. S2 Quality assessment of the studies included in systematic review and meta-analysis on the burden of MDR bacteria among HIV-positive individuals in Ethiopia.**

| Author, year of publication | Q1 | Q2 | Q3 | Q4 | Q5 | Q6 | Q7 | Q8 | Q9 | Total score (9%) |
| --- | --- | --- | --- | --- | --- | --- | --- | --- | --- | --- |
| Tilahun et al, 2023 | Y | Y | Y | Y | Y | Y | Y | Y | Y | 9 |
| Adhanom et al, 2019 | Y | Y | N | Y | Y | Y | Y | Y | Y | 8 |
| Genetu & Zenebe, 2020 | Y | Y | Y | Y | Y | Y | Y | Y | N | 8 |
| Ayele et al, 2020 | Y | Y | N | Y | Y | Y | Y | Y | Y | 8 |
| Alebachew et al, 2016 | Y | Y | N | Y | Y | Y | Y | Y | NA | 7 |
| Tilahun et al, 2023 | N | Y | Y | Y | Y | Y | Y | Y | Y | 8 |
| Tessema et al, 2020 | N | Y | Y | Y | Y | Y | Y | Y | Y | 8 |
| Abebe et al, 2014 | N | Y | N | Y | Y | Y | Y | Y | Y | 7 |
| Gebre et al, 2022 | Y | Y | Y | Y | Y | Y | Y | Y | NA | 8 |
| Bayleyegn et al, 2021 | Y | Y | Y | N | Y | Y | Y | Y | NA | 7 |
| Jemal et al, 2020 | Y | Y | Y | NA | Y | Y | Y | Y | Y | 7 |
| Manilal et al, 2019 | Y | Y | Y | Y | Y | Y | NA | Y | Y | 8 |
| Dadi et al, 2021 | Y | Y | Y | Y | Y | Y | Y | Y | Y | 9 |
| Fenta et al, 2016 | Y | Y | Y | NA | Y | Y | Y | Y | Y | 8 |
| Simeneh et al, 2022 | Y | Y | Y | Y | Y | Y | NA | Y | NA | 7 |
| Hantalo et al, 2020 | Y | Y | Y | NA | Y | Y | Y | Y | Y | 8 |
| Muhaba et al, 2022 | Y | Y | NA | NA | Y | Y | Y | Y | Y | 7 |
| Seid et al, 2020 | Y | Y | Y | Y | Y | Y | Y | Y | Y | 9 |
| Mulu et al, 2018 | Y | Y | Y | Y | Y | Y | Y | Y | Y | 9 |
| Adisu et al, 2023 | Y | Y | Y | N | Y | Y | Y | Y | Y | 8 |
| Zike et al, 2024 | Y | Y | Y | N | Y | Y | Y | Y | Y | 8 |
| Mitiku et al, 2023 | Y | Y | Y | N | Y | Y | Y | Y | Y | 8 |

**Key:** **Y** = Yes; **N** = Not reported, **NA** = Not appropriate

**Question codes:**

1. Was the sample frame appropriate to address the target population?

2. Were study participants sampled in an appropriate way?

3. Was the sample size adequate?

4. Were the study subjects and the setting described in detail?

5. Was the data analysis conducted with sufficient coverage of the identified sample?

6. Were valid methods used for the identification of the condition?

7. Was the condition measured in a standard, reliable way for all participants?

8. Was there appropriate statistical analysis?

9. Was the response rate adequate, and if not, was the low response rate managed appropriately?
