# Supplementary material for: Burden of multidrug-resistant bacteria among HIV-positive individuals in Ethiopia: A systematic review and meta-analysis
Source: PLoS One. 2024 Aug 26;19(8):e0309418. doi: 10.1371/journal.pone.0309418 (PMC11346931; doi:10.1371/journal.pone.0309418)
Supplement: S1 Fig — (DOCX) [file pone.0309418.s003.docx]

Fig. S3 **Forest plots showed the pooled prevalence of MDR by each bacterial species among HIV-positive individuals in Ethiopia.**
